# Accelerated atherosclerosis in premenopausal women with rheumatoid arthritis – A 15-year follow-up study

**DOI:** 10.17305/bjbms.2020.5176

**Published:** 2021-08

**Authors:** Metka Koren Krajnc, Radovan Hojs, Iztok Holc, Željko Knez, Artur Pahor

**Affiliations:** 1 Division of Internal Medicine, Department of Rheumatology, Maribor University Medical Centre, Ljubljana Slovenia; 2 Medical Faculty, University of Maribor, Maribor, Slovenia; 3 Division of Internal Medicine, Department of Nephrology, Maribor University Medical Centre, Maribor, Slovenia; 4 Faculty of Chemistry and Chemical Engineering, University of Maribor, Maribor, Slovenia

**Keywords:** Rheumatoid arthritis, atherosclerosis, cardiovascular disease, metalloproteases

## Abstract

Rheumatoid arthritis (RA) is a chronic inflammatory disease associated with increased mortality and morbidity due to the higher risks for cardiovascular disease. Traditional risk factors are insufficient to predict accelerated atherosclerosis in RA patients. The aim of this long-term prospective study was to investigate the relationships of asymptomatic atherosclerosis with traditional risk factors as well as inflammatory markers in patients with RA and matched healthy controls. Laboratory test results, concentrations of inflammatory mediators, matrix metalloproteases (MMP) and inflammation markers in a total of 70 (60 at follow-up) premenopausal healthy women with RA and 40 (34 at follow-up) matched controls were compared. B-mode ultrasound was applied for imaging of the carotid arteries for detection of asymptomatic atherosclerosis. Correlations with different factors were evaluated. The expression levels of intercellular adhesion molecules, vascular cell adhesion molecules (VCAMs), interleukin 6, tumour necrosis factor alpha and MMP-3 were significantly higher in the patient group during the follow-up period. The extent of plaque formation was greater in the patient group as compared to that in the control group (42.4% vs. 12.9%, respectively, *p* = 0.005), as was the cIMT (*p* = 0.001). By bivariate regression analysis, only VCAM expression was predictive of plaque formation (r = 0. 341, *p* = 0.016), but not for cIMT (r = −0.130, *p* = 0.327) in premenopausal female patients with RA at 15 years. These findings indicate that asymptomatic atherosclerosis is accelerated in premenopausal women with RA. During follow-up, there was an association between inflammation and accelerated atherosclerosis. Furthermore, VCAM was significantly correlated with plaque formation in RA patients.

## INTRODUCTION

Rheumatoid arthritis [RA] is a chronic autoimmune inflammatory disease that is associated with a two-fold greater risk of heart failure [1-7]. Although coronary artery disease is the primary cause of death, conventional cardiovascular (CV) risk factors may not fully account for the accelerated atherosclerosis in RA patients as compared to the general population [3-5]. Inflammation is the main culprit of RA and atherosclerosis, which share the same pathophysiological features and risk factors [1-7]. Although both are chronic inflammatory diseases, atherosclerosis affects the arterial wall and RA the synovial joint. Atherosclerosis evolves in several stages, during which adhesion molecules and pro-inflammatory stimuli play important roles in atheroma formation [2,7]. The inflammatory process of RA includes endothelial activation, expression of adhesion molecules and infiltration of the synovium with inflammatory cells, which begin in the synovial joints [2,7]. The pro-inflammatory cytokines tumour necrosis factor alpha (TNF-α) and interleukin-6 (IL-6), which are involved in the pathogenesis of RA, are predictive of subsequent CV events, as both play important roles in atherosclerosis [3,4,6]. Also, the expression levels of intercellular adhesion molecules (ICAMs), vascular cell adhesion molecules (VCAMs) and pro-inflammatory cytokines are elevated in RA, which results in altering of endothelial homeostasis in RA patients [2-4], leading to increased endothelial permeability and infiltration of lipids into the arterial wall, and subsequent formation of atherosclerotic plaques [3].

Inflammatory cytokines, such as interleukin-1 (IL-1) and TNF-α, also stimulate the production of matrix metalloproteinases (MMPs), which are enzymes that are able to degrade the extracellular matrix [8]. In the pathogenesis of RA, MMPs degrade collagen, resulting in joint destruction. MMPs have also been implicated in the development of atherosclerosis from the initial lesion to plaque rupture due to destruction of the vascular extracellular matrix [9].

Chronic inflammation, manifested by persistently increased C reactive protein (CRP), is associated with subclinical atherosclerosis and CV-realted death [10]. The results of a Spanish study suggested that CV events and related deaths are associated with chronic inflammation and genetic predisposition [11].

CV risk, which has been correlated with the extent of inflammation, is considered an independent risk factor for the severity of RA [1,4]. It is speculated that better control of RA could suppress CV disease [1,4-6].

Recent recommendations of the European League Against Rheumatism (EULAR) emphasise the importance CV risk management to reduce inflammation in RA [12,13]. The EULAR reported that traditional CV risk factors should be multiplied by a factor of 1.5 for management of RA [12]. Recent studies by Gualtierotti et al. [14,15] demonstrated that specific risk calculators underestimate the CV risk for RA. The increased risk of CV disease associated with RA is the result of complex interactions of chronic inflammation, traditional CV risk factors and genetic components; therefore, it is important to identify additional markers [16]. The authors suggested the creation of a consistent set of genetic markers and serum levels of adipokines and biomarkers to improve CV risk stratification of RA patients.

Paradoxically, methotrexate has been shown to reduce the risk of CV disease [2,15], despite increasing homocysteine levels, which is associated with an increased risk for CV diseases [14,15]. Biologic therapies to suppress inflammation, which is crucial for atherosclerosis development and plaque formation, have been shown to effectively reduce and prevent CV disease [4-7,17], thereby highlighting the need for early therapeutic interventions.

Carotid ultrasound is the most widely accepted non-invasive imaging method to assess asymptomatic atherosclerosis, carotid intima-media thickness (cIMT) and plaque formation [18,19], and is one of the best predictors of major CV events for CV risk stratification in RA [1]. Plaque formation and cIMT have been applied as surrogate markers for the prognosis of subclinical atherosclerosis [3].

The aim of this long-term prospective study was to identify potential risk factors of atherosclerosis associated with cIMT and plaque formation in women with RA in comparison to healthy female RA patients at low risk for CV disease. The association between markers of atherosclerosis and inflammation were based on data collected at baseline and after 15 years.

## MATERIALS AND METHODS

The cohort of this prospective observational study included 70 premenopausal, but otherwise healthy, women who were diagnosed with RA at baseline in accordance with the guidelines of the American College of Rheumatology diagnostic criteria and 40 age- and sex-matched healthy controls recruited from the Outpatient Clinic of the Maribor University Medical Centre. Data of both groups were collected at baseline and after 15 years [20-22]. Patients with a history of hypertension, diabetes, myocardial infarction, coronary artery disease and stroke were excluded from analysis. Clinical information, laboratory data and ultrasound of cIMT were obtained. Symptoms were evaluated at baseline and follow-up, while disease activity was assessed only in the follow-up study. Patients were treated by their own rheumatologist according to the EULAR guidelines.

Blood samples were collected in 2001 and 2016. Laboratory tests were performed with standardised sets for the sedimentation rate (ESR), lipid concentrations and expression levels of CRP, IL-6, interleukin-2R (IL-2R), TNF-α, ICAMs and VCAMs. The serum concentrations of MMP-3 and MMP-9 were measured with a multiplex enzyme-linked immunosorbent assay and confirmed by high-performance liquid chromatography/tandem mass spectrometry [23] at the time of the follow-up study, since necessary equipment was not available at the time of the baseline study. Most laboratory tests were performed at the Maribor University Medical Centre, as serum MMP-3 and MMP-9 levels were assayed at the Faculty of Medicine of the University of Maribor. The use of medications was documented. The cIMT was measured and plaque formation was assessed by B-mode ultrasonography using a linear 10-MHz probe at both baseline and follow-up. All measurements were performed by the same experienced investigator. All examinations were performed with the patients lying in the supine position with the head resting comfortably and the neck hyperextended and rotated away from imaging transducer. Both carotid arteries were scanned within 2 cm proximal to the dilatation of the carotid bulb and digitised still images were obtained. The cIMT and extent of plaque formation of the common carotid arteries and the proximal internal carotid artery were recorded.

### Ethical statement

All subjects provided signed informed consent. The study protocol was approved by the Ethics Committee of Slovenia (Slovenian Ethics Committee) and conducted in accordance with the ethical principles for medical research involving human subjects of the Declaration of Helsinki.

### Statistical analysis

All data analyses were conducted using IBM SPSS Statistics for Windows, version 24.0. (IBM Corporation, Armonk, NY, USA). Quantitative variables are presented as the mean ± standard deviation. The distribution of the data was assessed with the Kolmogorov–Smirnov test. Qualitative variables are presented as the frequency and percentage. Difference between normally distributed independent variables were compared using the *t*-test, while differences in qualitative variables between groups were assessed using the c² test. Bivariate linear regression analysis was used to test the associations between cIMT and plaque formation and numerical variables. Those with statistical difference between the groups were then selected. A probability (*p*) value of < 0.05 was considered statistically significant.

## RESULTS

Of the 70 patients recruited in 2001, six had died and four were lost to follow-up. The causes of deaths were cancer in four patients and septic shock in two. No deaths were due to a CV event. Three patients had a CV event (acute myocardial infarction) during the study period and all survived without major consequences. Sixty (85.7%) of the 70 patients and 34 (85.0%) of the 40 healthy controls completed the study. The most common reason for those in the control group to drop out of the study was relocation to another city. There were no deaths or CV events in the healthy control group.

Of the 64 surviving patients, 55 (85.6%) were taking disease-modifying antirheumatic drugs (DMARDs) within the 15-year study period, which included conventional synthetic DMARDs (i.e., methotrexate, sulphasalazine, leflunomide or chloroquine; collectively, 45.3%) or biologic DMARDs, such as anti-TNF-α, tocilizumab and rituximab (16.4%, 10.9% and 9.1%, respectively). Only 17 (30.9%) patients were taking low-dose glucocorticoids as well (Table 1). At baseline, none of the patients were taking biologic DMARDs.

**TABLE 1:**
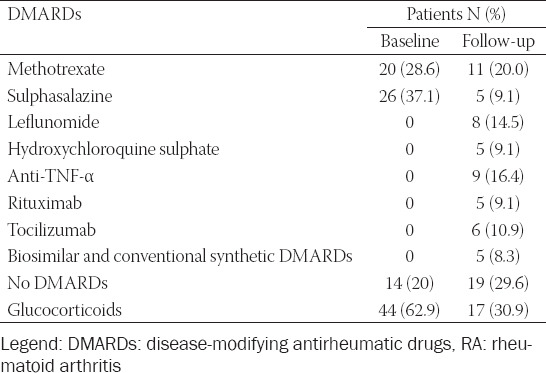
DMARDs used for treatment of RA

During the follow-up period, ten patients developed arterial hypertension (all treated with angiotensin-converting enzyme inhibitors), one developed insulin-dependent diabetes and four developed dyslipidaemia (all treated with statins).

In the follow-up study, the mean Disease Activity Score-28 for Rheumatoid Arthritis was 3.37 ± 1.31.

The patient group had statistically significant more plaques at baseline and follow-up. At baseline, plaque formation was detected in 7 (10%) women in the patient group, but none in the control group (Table 2). After 15 years, plaque formation was detected in 25 (42. 4%) women in the patient group and 4 (12. 9%) in the control group (Table 2). The difference in plaque formation from baseline to 15 years was statistically significant (*p* = 0.005).

**TABLE 2:**
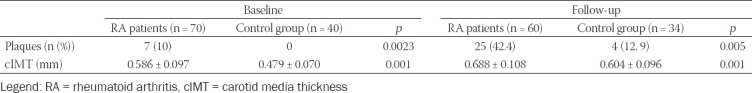
Number (percent) of plaques and cIMT at baseline and follow-up

The cIMT at baseline was statistically significantly higher in the patient group. In addition, the difference was significant even after 15 years of follow-up (*p* = 0.001) (Table 2).

As shown in Table 3, there were no significant differences in traditional risk factors for RA between the patient and control groups at baseline and after 15 years of follow-up.

**TABLE 3:**
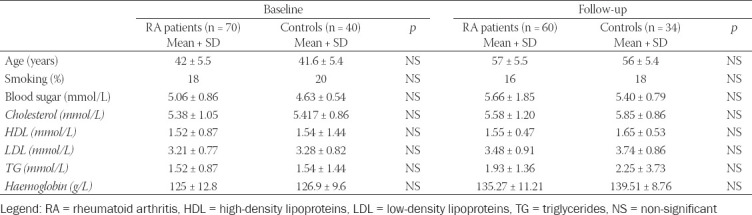
Traditional risk factors in RA patients and control subjects at baseline and follow-up

Serum levels of CRP, ICAMs, VCAMs, IL-6 and TNF-α and the ESR were significantly higher in the patient group (Table 4).

**TABLE 4:**
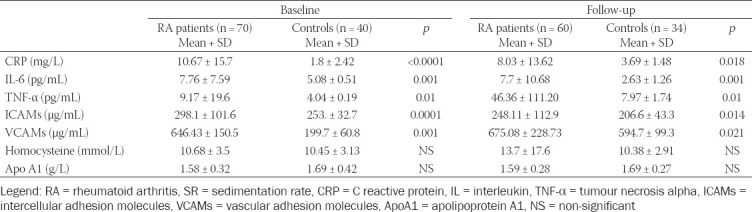
Inflammatory markers in RA patients and control subjects at baseline and follow-up

Serum levels of MMP-3 and MMP-9 were higher in the control group than the patient group (13.09 ± 17.06 vs. 26.27 ± 10.62 and 225.51 ± 136.29 vs. 246.74 ± 170.11, respectively, *p* = 0.000 and 0.511), but only MMP-3 levels were significant higher.

There was no association between the traditional risk factors (glucose levels, hypertension and dyslipidaemia) and asymptomatic atherosclerosis (cIMT and plaques) in the patient and control groups. In addition, there was generally no correlation between inflammatory markers and asymptomatic atherosclerosis, with the exception of VCAMs and plaque formation (r = 0.341; *p* = 0.016) in the patient group.

## DISCUSSION

The results of the present study showed that the atherosclerotic process was accelerated in otherwise healthy premenopausal female RA patients without other traditional risk factors as compared to heathy controls. Measurements of cIMT and plaque formation were used as surrogate markers of asymptomatic atherosclerosis. Differences were evident even at baseline, as plaque formation had already occurred in the carotid arteries of some of the women in the patient group. In addition, the cIMT value was significantly higher in the patient group at baseline. At follow-up, this difference was even greater.

The clinical relevance of these findings is even more important in consideration of previous studies, which reported that the risk of acute myocardial infarction increases by 43% for every 0.163-mm increase in cIMT and that the prevalence of carotid plaque is an even more reliable predictor of CV events [1,13,24-27]. A recent 5-year-prospective follow-up study conducted by Corrales et al. [28] of 327 RA patients reported that 23 (7.0%) had died and 27 (8.3%) had experienced CV events, indicating that the presence of carotid plaques was predictive of the development of CV events and death in RA patients. The authors also reported that the best predictor of CV risk was the presence of carotid plaques.

Inflammation seems to be the most important risk factor for accelerated atherosclerosis in RA patients. However, in the present study, there were no correlations between traditional risk factors and cIMT or plaques as predictors of atherosclerosis. Recent evidence has clearly suggested that genetic factors may play important roles in CV risk in RA. A study by Lopez-Mejias et al. [29] identified a genetic variation of RARB gene that contributes to the development of subclinical atherosclerosis in RA patients and may represent a turning point for better understanding of the underlying pathogenic mechanisms.

Several studies have proposed common underlying pathogenic mechanisms of atherosclerosis and inflammation [14,27,30]. Accelerated atherosclerosis in RA is initially due to the presence of endothelial dysfunction, as injury to the vascular endothelium is the primary event in atherosclerosis, and systemic inflammation has been linked to endothelial dysfunction [30].

Expression levels of adhesion molecules and cytokines are purportedly higher in RA patients, suggesting that inflammation could be an independent risk factor [4,7,14]. Systemic inflammation is common to the pathogenesis of both RA and atherosclerosis [1,31,32]. In addition, VCAM expression was significantly correlated with plaque formation in the present study, indicating that inflammation might be an important factor in the development of atherosclerosis. Davies et al. [33] stated that VCAM is a significant predictor of CV risk in RA patients, as VCAM levels were correlated with disease activity, indicating potential use as biomarkers of CV disease in RA patients. Santos et al. [34] suggested that circulating VCAM levels might be predictive of CV disease, as higher levels were found to increase CV-associated mortality and were predictive of future CV events. Furthermore, Castro et al. [24] proposed ESR and ICAM as independent predictors of CV events. In the present study, ICAM levels were significantly elevated in RA patients, but there was no correlation between ICAM levels and asymptomatic atherosclerosis in RA patients. In addition, ESR and CRP levels were significantly higher in the patient group, thereby supporting the idea that inflammation is a major risk factor for asymptomatic atherosclerosis in RA patients. In a previous study, CRP expression was correlated with cIMT [20]. However, as mentioned above, there were no differences in traditional risk factors for atherosclerosis between the patient and healthy control groups.

Notably, in the present study, MMP levels were higher in the healthy control group. Isik et al. [34] examined the implications of MMP-9 in the development of accelerated atherosclerosis, but found no correlation between MMP-9 serum levels and cIMT or active inflammation, and concluded that MMP-9 is not a reliable predictor of disease severity or accelerated atherosclerosis in RA patients, possibly because of the effect of immunomodulatory therapy on MMP expression. MMP-9 plays important roles in the later stages of atherosclerotic plaque formation, particularly in plaque rupture [3]. However, in the present study, there were no correlations between asymptomatic atherosclerosis and MMP-3 or MMP-9 expression levels. As a possible explanation for the lower levels of MMP-9 in the present study, only young women without risk factors for CV disease were enrolled. The cytokine TNF-α is very important in pathogenesis of RA by reducing inflammation and, subsequently, the risk of atherosclerosis [1]. A prior meta-analysis reported that anti-TNF-α therapy is associated with increased levels of high-density lipoprotein cholesterol, triglycerides and total cholesterol in RA patients [7]. A previous study by our group confirmed a correlation between TNF-α and cIMT in the patient group [23].

As stated in many studies, chronic inflammation should be considered a modifiable risk factor, such as smoking, obesity and the lack of physical activity [6,7,18,25].

Dyslipidaemia is common in RA. Statins, which are used to lower lipids, have also showed some anti-inflammatory properties with even greater effects when combined with DMARDs [34]. Atherosclerosis is currently viewed as an inflammatory disease and modification of lipoproteins during inflammation was reported to accelerate atherogenesis [13]. Our group previously reported a correlation between triglyceride levels and cIMT, indicating the importance of lipids in asymptomatic atherosclerosis in RA patients [20].

During the follow-up period of the present study, there was no significant difference in lipid levels between the patient and healthy control groups. It is known that cholesterol levels are decreased in the presence of active inflammation [25,35]. Toms et al. [36] observed lower levels of cholesterol and low-density lipoprotein cholesterol (LDL) in patients with active RA. In the present study, disease activity was moderated in the patient group, which could explain the lack of significant differences in cholesterol and LDL levels between the patient and control groups.

Methotrexate is a cornerstone therapy for RA and a first-choice immunomodulatory drug. Many studies have reported that methotrexate therapy reduced mortality, despite the increased homocysteine levels [14,15]. In the present study, there was no correlation of methotrexate and other DMARDs with accelerated atherosclerosis. The same was true in the group taking low-dose glucocorticoids. These findings are probably due to the small group sizes and even smaller sample sizes taking different medications for RA.

The findings of the present study are consistent with epidemiologic data demonstrating the increased CV risk, which is often underestimated, in RA patients as compared to the general population [37-40]. Carotid ultrasound is very sensitive for the detection of subclinical atherosclerosis in RA patients [41]. The results of previous studies suggest that additional assessment by carotid ultrasound may be useful to establish the actual CV risk in RA patients [41,42].

There were some limitations to this study that should be addressed. First, this was a single-centre study of a relatively small number of patients and healthy controls. Second, the patient cohort was limited to only women. Therefore, further studies of both men and women are needed to better apply these results in everyday practice because of the known sex differences in hormone status and, consequently, atherosclerotic risk. On the other hand, the strengths of this study were the inclusion of a unique group of premenopausal women without traditional risk factors for atherosclerosis and the long-term (15 years) observation period, which is the longest reported to date in the literature.

## CONCLUSION

Inflammation risk factors seem to be more important than traditional risk factors for the atherosclerotic process in young premenopausal females with RA. VCAM expression levels were significantly associated with atherosclerotic plaque formation. Therefore, even premenopausal women with RA should be properly and regularly managed for CV risk and aggressively treated for RA because chronic inflammation is also a risk factor.
